# Revealing 3D magnetization of thin films with soft X-ray tomography: magnetic singularities and topological charges

**DOI:** 10.1038/s41467-020-20119-x

**Published:** 2020-12-14

**Authors:** A. Hierro-Rodriguez, C. Quirós, A. Sorrentino, L. M. Alvarez-Prado, J. I. Martín, J. M. Alameda, S. McVitie, E. Pereiro, M. Vélez, S. Ferrer

**Affiliations:** 1 SUPA, School of Physics and Astronomy, University of Glasgow, Glasgow, G12 8QQ UK; 2 Depto. Física, Universidad de Oviedo, 33007 Oviedo, Spain; 3 CINN (CSIC – Universidad de Oviedo), 33940 El Entrego, Spain; 4 ALBA Synchrotron, 08290 Cerdanyola del Vallès, Spain; 5 Present Address: Depto. Física, Universidad de Oviedo, 33007 Oviedo, Spain

**Keywords:** Materials science, Condensed-matter physics, Nanoscale materials, Techniques and instrumentation, Nanoscience and technology

## Abstract

The knowledge of how magnetization looks inside a ferromagnet is often hindered by the limitations of the available experimental methods which are sensitive only to the surface regions or limited in spatial resolution. Here we report a vector tomographic reconstruction based on soft X-ray transmission microscopy and magnetic dichroism data, which has allowed visualizing the three-dimensional magnetization in a ferromagnetic thin film heterostructure. Different non-trivial topological textures have been resolved and the determination of their topological charge has allowed us to identify a Bloch point and a meron-like texture. Our method relies only on experimental data and might be of wide application and interest in 3D nanomagnetism.

## Abstract (web summary)

Although magnetic tomography has been used in the past to determine the 3D magnetization of materials its application to thin films remains challenging. Here the authors reconstruct the magnetization of a thin film, enabling the measurement of topological charges of magnetic singularities.

## Introduction

Due to the importance of domains in the magnetic properties of materials, including thin films or nanostructures for applications in spintronics, magnetic domain visualization methods have been an active field of research in the last decades (for a recent review, see refs. ^1,2^). Transmission methods are excellent for the visualization of magnetic states in the sample and require probes with high enough penetration depths. For instance, neutron radiographies have allowed imaging the interior of ferromagnets at sub-millimeter scale^3,4^. Transmission electron microscopy may probe film thicknesses up to 100 nm^5^, which has been used to characterize in-plane magnetization by electron holography^6^ and to tomographically reconstruct the magnetic vector potential of individual nanoelements^7^. More recently, the magnetic configuration within a 5-µm diameter GdCo_2_ micropillar has been reconstructed by magnetic vector ptychotomography using hard X-rays (wavelength of 0.17 nm)^8^. This has been the first experimental realization of a complete tomographic reconstruction using the different angular projections to recover the three-dimensional (3D) magnetization configuration. The same method has been applied to visualize magnetization dynamics^9^. In the soft X-ray range, reflection and transmission experiments have reported interesting results^10–14^. X-ray magnetic scattering has allowed a detailed characterization of skyrmions in periodic arrays^10,11^ and of periodic striped domains in NiFe/CoPd multilayers^12^. In transmission, two-dimensional (2D) magnetization patterns have been extracted from tubular samples acquired at different angles^13^. This approach was based on exploiting the small thickness of the tubular shell to recover, via a single angular series around a particular rotation axis, the magnetic state of the system.

Tomographic imaging of extended thin samples is of particular difficulty due to their high aspect ratio (lateral dimensions » thickness, i.e. millimeters vs. few hundreds of nanometers). This has hindered a direct 3D visualization of the magnetization in thin films and multilayers of arbitrary magnetization configuration and it is a major limitation since most magnetic devices are fabricated on substrates with macroscopic lateral dimensions. Therefore, a variety of basic and application-related topics such as the thickness dependence of magnetic textures in chiral multilayers^15^, or the optimization of spin torque oscillators^16^, have been addressed indirectly^15–21^ by image simulations and clever sample designs. Thus, magnetic vector tomography in extended thin films can become an essential tool for the evolution of nanomagnetism from 2D configurations to the complex 3D magnetization textures and structures explored nowadays^2^ including magnetic hopfions^22^ and skyrmion dots^23^.

Here, we present the first results of a 3D tomographic reconstruction of a magnetic thin film heterostructure, 240 nm thick, using transmission soft X-ray microscopy and a recently developed vector reconstruction method^21^. This combination revealed the 3D magnetization vector **m** in an arbitrary configuration and without any a priori assumption. A three-layer thin film having identical top and bottom layers allowed us to test our method and identify their differences and magnetic singularities. The reconstructed magnetization enabled to evaluate the topological charge within the film providing a powerful tool for precise quantitative characterization of magnetic singularities, especially for 3D magnetic structures.

## Results

### Magnetic tomography with X-rays

X-ray magnetic circular dichroism (XMCD) provides the contrast mechanism that allows revealing the vector nature of the magnetization: the dichroic effect of a volume element in the material depends on the dot product **σ**•**m** of the spin angular momentum of the circularly polarized photons **σ** (parallel/antiparallel to the propagation direction for clockwise (CW) or counter-clockwise (CCW) polarizations) and the local magnetization **m**^24^. By exploiting the angular dependence of **σ**•**m** and using appropriate algorithms, we can obtain the vector reconstruction of the magnetization. In short, the reconstruction method is based on processing two orthogonal tomograms formed by transmission X-ray projections of the sample acquired under different angular orientations. The intensity recorded at each pixel of the detector depends on the line integral of **σ**•**m** along a specific X-ray path through the sample. By creating a volume model composed of voxels, the line integral can be discretized into a linear equation where the unknowns are proportional to the magnetization vectors within each voxel along the X-ray path. A system of linear equations is then constructed considering all the pixels and all the different projections. The iterative method Algebraic Reconstruction Technique is used to solve the system of equations^25^ which allows us to obtain the magnetization vectors. Limitations of the method arise from the extended nature of the film and from the experimental setup: The effective film thickness increases at grazing angles which prevents photon transmission and geometrical shadowing effects, coming from the support used, limit the measurable angular range (in this case ±55°) causing the so called “missing wedge” (details in Methods and Supplementary Information). Finally note that, while magnetic tomography is based on magnetic absorption contrast, X-ray diffraction effects from periodic magnetic patterns may also occur although they are ignorable in our case as discussed in the Supplementary Information.

### Magnetic trilayer and X-ray microscope

**Fig. 1:**
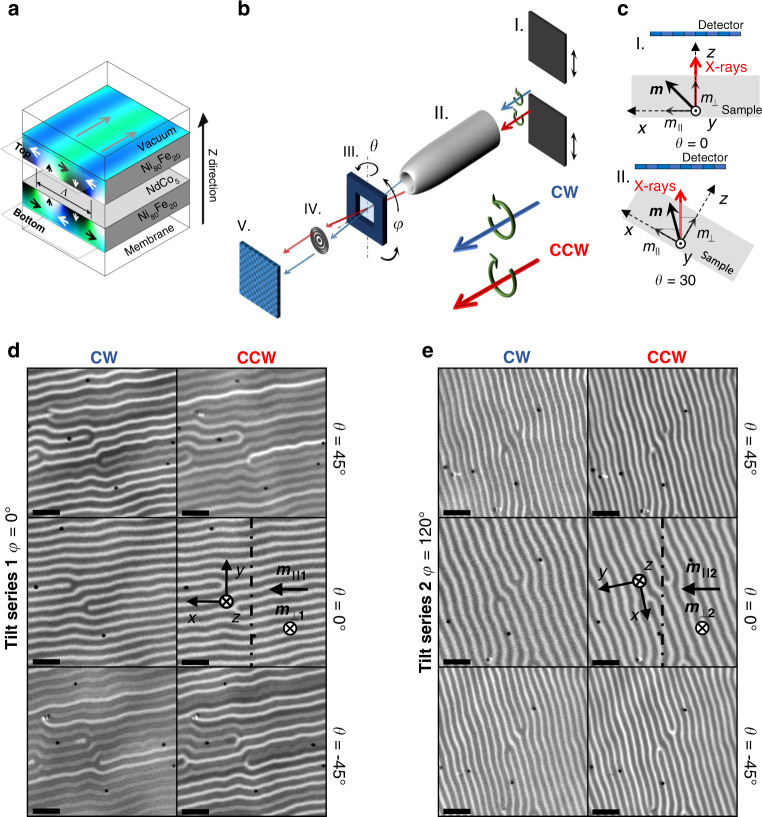
Sample sketch, experimental set up, and Tilt series image data. **a** Sketch of magnetization in the striped domain pattern in NiFe/NdCo/NiFe trilayer. **b** Scheme of the full-field magnetic soft X-ray transmission microscope: (I) ancillary slits to select the polarization of the X rays (circular CW or CCW), (II) capillary condenser, (III) goniometric stage (*θ* rotation), *φ* rotation manually performed outside the microscope, (IV) Fresnel zone plate, and (V) CCD detector. **c** Top view sketch to illustrate sample reference frame and magnetization components probed during a *θ* rotation (around the *y* axis): (I) Normal incidence *(θ* = 0°) leads to out-of-plane (*m*_⊥_) sensitivity. (II) Oblique incidence (*θ* = 30°) allows for in-plane (*m*_||_) and out-of-plane (*m*_⊥_) sensitivity. **d**, **e** Transmittance images for two different sample configurations, incidence angles, and photon polarizations: **d** Tilt series 1 (*φ* = 0°), **e** Tilt series 2 (*φ* = 102°). Reference frame indicated in both datasets. Probed magnetization components (*m*_||_, *m*_⊥_) indicated for each configuration. Dot–dash vertical line indicates *θ* rotation axis. Scale bars 1 μm. The black dots in the images of **d** and **e** are Au fiducials for image alignment.

We fabricated a magnetic trilayer Ni_80_Fe_20_(80 nm)/NdCo_5_(80 nm)/Ni_80_Fe_20_(80 nm) on a silicon nitride membrane of 50 nm. The NdCo_5_ layer^26^ displays weak perpendicular magnetic anisotropy (PMA) leading to magnetic striped domain patterns with canted up and down magnetizations^27^. The exchange interaction imprints the central striped pattern on the magnetically soft Ni_80_Fe_20_ films (Permalloy: Py = Ni_80_Fe_20_), as sketched in Fig. 1a with blue and green color bands. The up and down magnetic striped domains have periodicity *Λ* and are separated by regions with in-plane magnetization depicted by the gray arrows on top of the sketch. The white and black arrows illustrate the magnetization pattern of the closure domains. X-rays tuned at the Fe L_3_ energy (wavelength 1.754 nm) were chosen to probe only magnetic domains at the top and bottom layers, which are nominally of identical atomic composition and cannot be distinguished by element specific 2D X-ray transmission microscopy^20,28^). A 3D method with in-depth sensitivity is necessary to separate their magnetizations and to characterize magnetic singularities within the striped domain pattern. Since no magnetic signal is originated from the central NdCo_5_ layer, it is represented as an empty space in the figure.

Figure 1b illustrates the main parts of the full-field X-ray transmission microscope of the Mistral beamline at the Alba synchrotron^29^. By acquiring images with circularly polarized X-rays with different handedness (CW or CCW) and at different rotation angles, the magnetization of the Py in a plane perpendicular to the rotation axis (*θ* rotation) can be determined. In the *x*, *y*, *z* sample reference frame (Fig. 1c), both the in-plane (*m*_||_) and the out-of-plane (*m*_⊥_) components of the magnetization can be detected. To probe the magnetization along the *y* axis that is not sensed in Fig. 1d (dot-dashed line), the sample was manually rotated from *φ* = 0° (panel d) to *φ* = 102° (panel e) where the *y* axis is nearly perpendicular to the rotation axis (dot-dashed line). We acquired images at angles *θ* ranging from −55° to 55° in 87 steps for each polarization and both azimuthal *φ* angles. We define the set of images with *φ* = 0° and *φ* = 102° as Tilt series 1 and 2, respectively. As seen in Fig. 1d, e, the images display the characteristic striped pattern of these multilayers. Note that the partially inverted magnetic contrast at −45° and +45° at each polarization results from the different signs of the products **σ**∙**M** $\propto \left( {m_{||}\sin \left( \theta \right) + m_ \bot \cos (\theta )} \right)$. The images show bifurcations of some stripes appearing with white, gray, or black contrasts depending on the X-ray helicity and *θ* angle. Bifurcations deserve particular attention, and will be later discussed in detail, since they are the key actors in the in-plane magnetization inversion and can act as nucleation centers of magnetic singularities^28,30,31^. The images in Fig. 1 have a magnetic contribution and a non-magnetic one arising from the absorption of the X rays by the electrons of the sample. To isolate the magnetic part, CW and CCW images were subtracted for the same *θ* angle. Details on image processing and alignment for obtaining the stack of images required for the tomographic reconstruction are provided in the Methods section and in Supplementary Information.

In what follows, we will first discuss the configuration of the domain walls by separately analyzing both tilt series, and later we will describe the magnetic singularities and determine their topological charge by analyzing both tilt series together.

### Tomographic reconstruction of magnetization configuration

**Fig. 2:**
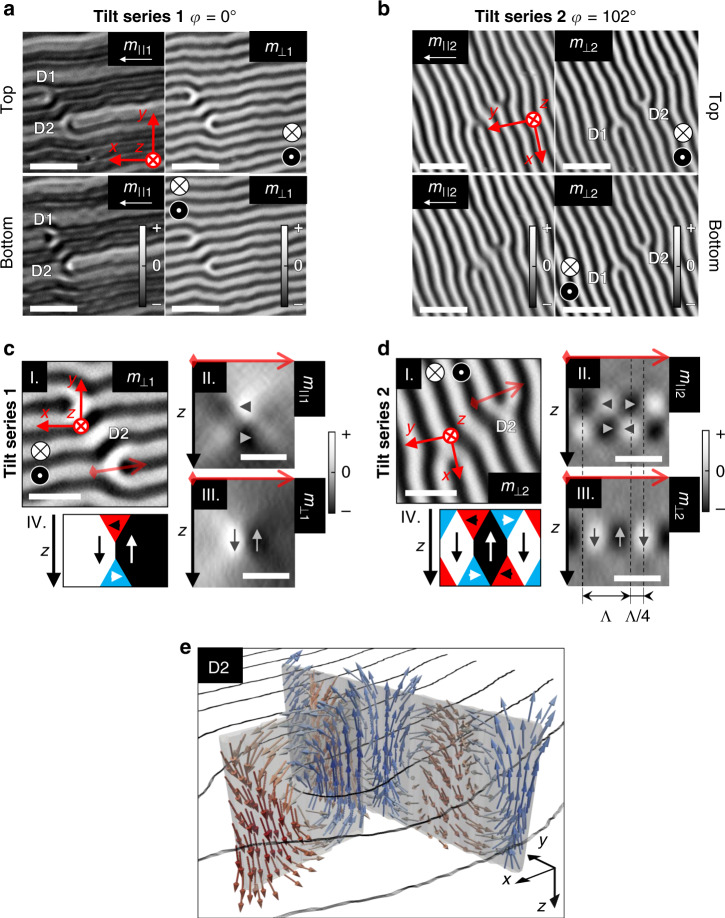
Tomographic reconstruction of 3D magnetization configuration in the striped domain pattern. **a**, **b** Reconstructed magnetization at the central slices of top and bottom Py layers showing in-plane (*m*_||_) and out-of-plane (*m*_⊥_) components from Tilt series 1(**a**, *φ* = 0°) and 2 (**b**, *φ* = 102°). Scale bars 1.4 µm. D1 and D2 index the bifurcations in the image. **c**, **d** Analysis of the reconstructed magnetization around dislocation D1 for Tilt series 1 (**c**) and 2 (**d**): (I) Top view of *m*_⊥_ at central slice of volume model. Red arrows indicate the extracted cross-sections. Cross-sections show in-plane (II, *m*_||_) and out-of-plane (III, *m*_⊥_) components of the reconstructed magnetization for Tilt series 1 (**c**) and 2 (**d**). (IV) Sketches showing the closure magnetization structure in the selected cross-sections for Tilt series 1 (**c**) and 2 (**d**). Scale bars 700 nm (top view) and 350 nm (cross-section). Gray scale bars indicate the sign of the magnetization. Vertical dashed lines in **d** indicate the striped domain pattern periodicity (*Λ*) and the *Λ*/4 dephasing in between *m*_||2_ and *m*_⊥2_ components in the closure domain structure. **e** 3D vector representation of the reconstructed magnetization from the selected cross-sections. Black lines indicate the Bloch domain-walls.

The tomographic reconstruction of XMCD data results in a 3D dataset of *m*_||_ and *m*_⊥_ within a 2625 nm × 2625 nm × 840 nm volume for Tilt series 1 and 2. A general view of the reconstructed magnetization at the individual top and bottom Py layers is shown in Fig. 2a, b. We observe that the in-plane magnetization *m*_||1_ (which is almost parallel to the striped domains) presents broad dark and bright regions parallel to the *x* direction corresponding to groups of striped domains with opposite average in-plane magnetization sense. Within them, an oscillatory contrast of thin wrinkles is also observed corresponding to the oscillation of the magnetization along the *y* direction from in-plane to out-of-plane within each striped domain (Fig. 2a). Clear differences appear in *m*_||1_ between the top and bottom reconstructed slices in the vicinity of the bifurcations (D1 and D2). The out-of-plane magnetization components (*m*_⊥1_ in Fig. 2a and *m*_⊥2_ in Fig. 2b) probe the same component in both tilt series and display identical alternating contrasts. They form the characteristic parallel striped pattern of up/down domains with small transverse undulations^32^, virtually identical for the top and bottom slices. In addition, the in-plane magnetization component *m*_||2_ shown in Fig. 2b (which is aligned near the *y* axis direction) exhibits periodic bright–dark oscillations that indicate a periodic change of sign in the magnetic component transversal to the striped domains, again very similar at top/bottom Py layers (see Supplementary Information for further details).

Magnetization configuration across the thickness can be obtained by representing the cross-sections containing the *z* axis of the reconstructed datasets (see Fig. 2c, d for a selected region close to bifurcation D2). The first thing to note in the cross-sections taken across the bifurcation core in Fig. 2c is the different vertical configuration of the *m*_||1_ and *m*_⊥1_ domains: while *m*_||1_ changes sign between top/bottom layers (Fig. 2cII), the out-of-plane magnetization *m*_⊥1_ does not (Fig. 2cIII). At this point, we wish to mention a methodological issue. In our tomographic reconstruction, we do not impose any a priori knowledge, thus we do not set **m** = 0 in the central layer that is not sensed by the X rays. This and the axial resolution of the tomogram (estimated as 85 nm in the Supplementary Information) generate a continuous magnetization that is not zero in the central part as expected and can be observed, for example, in the cross-section displayed in Fig. 2cIII where magnetization *m*_⊥1_ is reconstructed through the whole thickness of the heterostructure. As will become clear later, to determine the values of the topological charges, it is not necessary to know precisely where they are located since their magnitudes rely on the flux of the vector fields that they generate across closed surfaces which can be relatively far from them. This continuity physically mimics that of the magnetization imposed by the exchange interaction across the whole sample and could allow us to identify magnetization textures even if they were located within the central magnetic layer. Now, if we take into account the magnetization sense resulting from the signs of the *m*_||1_ and *m*_⊥1_ domains in Fig. 2c, we obtain a circulating vortex around the bifurcation core, as sketched in Fig. 2cIV: from left to right, a *m*_⊥1_ positive striped domain evolves into a negative one through the core of the dislocation showing positive (negative) *m*_||1_ component at bottom (top) Py layers. A similar magnetization circulation across the thickness is observed in the cross-sections transverse to the striped domain pattern displayed in Fig. 2d: positive *m*_||2_ regions of the upper Py layer are on top of negative regions of the bottom layer (Fig. 2dII), whereas *m*_⊥2_ domains are continuous across the thickness with a lateral up/down oscillation of measured period *Λ* = 390 nm (Fig. 2dIII). There is a *Λ*/4 dephasing between the in-plane and out-of-plane magnetization oscillation (see dashed vertical lines Fig. 2dIII and Fig. 2dIV) resulting in the typical closure domain structure (or Neel caps (Supplementary Information)) characteristic of striped domain patterns (sketched in Fig. 2dIV).

By combining both cross-sections in vector representation, we obtain a good 3D view of the magnetization circulation across the thickness around dislocation D2 (see Fig. 2e where the black lines indicate the Bloch domain walls separating differently oriented striped domains). These results demonstrate the capability of the tomographic reconstruction to characterize complex 3D magnetization patterns.

The reconstructed signals shown in the cross-sections in Fig. 2 locate the magnetization in a *z*-extension of ∼300 nm, which agrees with the nominal thickness of the film (240 nm) considering the axial resolution in the *z* direction. However, the vertical confinement of the tomographic reconstruction in the *z* direction for thin film geometry is sensitive to parallax effects as discussed in the Supplementary Information.

### Magnetic singularities and experimental topological charge

**Fig. 3:**
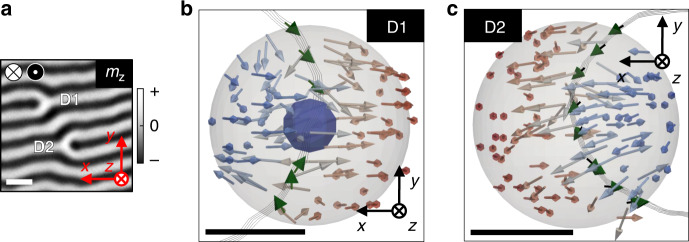
Tomographic reconstruction of magnetic singularities. **a** Out-of-plane (*m*_*z*_) component from the joint reconstruction of Tilt series 1 and 2 at the central slice of the volume model. Dislocations D1 and D2 are indicated. Gray scale bar indicates the sign of *m*_*z*_. **b**, **c** 3D representation of the magnetic textures at the cores of D1 (**b**) and D2 (**c**). Arrows depict the orientation of the magnetization colored with Blue-White-Red indicating Negative-Zero-Positive sign of *m*_*z*_. Green arrows show the magnetization within the Bloch domain-wall at the core of the dislocations. Blue sphere marks the Bloch-point singularity. Scale bar **a** 525 nm, **b** 75 nm, **c** 105 nm.

The joint reconstruction using the data from both Tilt series allowed us to obtain the 3D magnetization at the core of dislocations. This required careful alignment of the projections of both Tilt series facilitated by the gold fiducials that were added to the sample (visible in Fig. 1d, e as black dots) (details in Supplementary Information). Figure 3a shows the *m*_*z*_ component at the central slice of the reconstructed volume that has dislocations D1 and D2. Both defects look very similar in panel a, while clear differences become apparent in the vector representation in panels b and c. While bifurcation D1 displays a convergent in-plane magnetization toward the core of the defect in Fig. 3b (*m*_*x*_ keeps its negative sign and goes to zero while *m*_*y*_ has positive (negative) value at the lower (upper) branch of the Bloch domain-wall in the proximity of the dislocation core), bifurcation D2 exhibits a continuous CCW circulation following the domain-wall (Fig. 3c). The magnetic configuration at D2 resembles a ½ skyrmion with a 180° in-plane magnetization rotation (green arrows) and an out-of-plane polarity change from positive to negative *m*_*z*_ (blue/red arrows). This is consistent with a meron-like configuration with topological charge ½ typical of striped domain patterns^31,33^. On the other hand, Fig. 3b strongly suggests a circulating Bloch-point at the core of the dislocation^8,34,35^. The position of the Bloch point is depicted by the 40 nm diameter blue sphere in Fig. 3b that will be discussed below. The presence of Bloch-points at bifurcation cores is not surprising since they drive the in-plane magnetization reversal in striped domain patterns^28,30^.

**Fig. 4:**
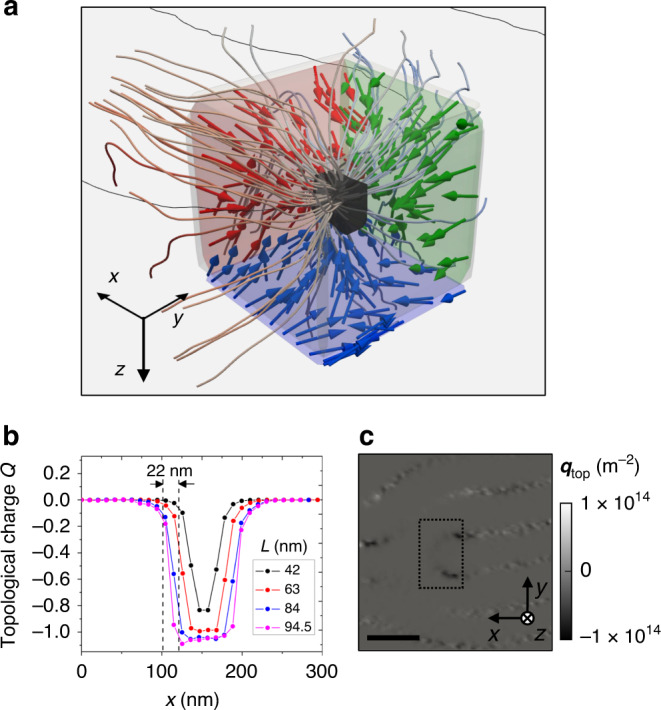
Experimental determination of topological charges. **a** Streamlines and arrows of emergent magnetic field around the Bloch point in bifurcation D1 enclosed by a cube of 100 nm lateral size. **b** *x* Profile of the total topological charge *Q* obtained integrating over cubes of different side dimensions indicated in the inset. The precision on the localization of the Bloch point is ca. 22 nm (see main text). **c** Density of topological charge *q*_top_ in the *x*, *y* plane (*q*_*z*_) at the central part of the sample near D2 of Fig. 3c. The dashed rectangle shows the integration area. Scale bar 250 nm.

A deeper insight on the nature of D1 and D2 can be achieved by evaluating their topological charge defined as^35,36^

1 $$Q = \frac{1}{{8\pi }}{\int} { \in _{ijk}} {\mathbf{m}} \cdot \partial _j{\mathbf{m}} \times \partial _k{\mathbf{m}}{\mathrm{d}}A_i = \frac{1}{{4\pi }}{\int} {q_i} {\mathrm{d}}A_i,$$

where *i*, *j*, *k* refer to the *x*, *y*, *z* coordinate axes, ∈_*ijk*_ is the Levi-Civita antisymmetric tensor, d*A*_*i*_ is an area element perpendicular to the *i* axis, and **m** is the unit magnetization. The integral extends over a closed surface *S* enclosing the singularity. From a theoretical point of view^36–38^, the magnetic singularity is characterized by the Berry curvature $q_i = \frac{1}{2} \in _{ijk}{\mathbf{m}} \cdot \partial _j{\mathbf{m}} \times \partial _k{\mathbf{m}},$ which is a pseudovector field proportional to an “emergent” magnetic field $B_i^e = \hbar q_i$. The integral of **B**^*e*^ over *S* is proportional to a quantized topological charge and quantized flux. Topological charges have analogies with electrical charges that are worth mentioning here. A point electrical charge creates an electric field **E** that has zero divergence everywhere except at the position of the charge. The same occurs with the emergent field **B**^*e*^ created by a topological charge. In both cases, the magnitudes of the charges can be determined by evaluating the flux of the corresponding fields through any arbitrary closed surface that encloses the charges. If the positions of the charges are imprecise, it suffices to select a sufficiently large integration surface. This is what has been done in Fig. 4.

In the literature, the most commonly investigated magnetic singularities are skyrmions, merons, and vortices, which are 2D singularities since the magnetization is defined in a plane *x*, *y*, and $\partial _z{\mathbf{m}} = 0$. As a consequence, Eq. (1) simplifies to (1/4π)*∫*${\boldsymbol{m}} \cdot \partial _x{\boldsymbol{m}} \times \partial _y{\boldsymbol{m}}\,{\mathrm{d}}x\,{\mathrm{d}}y$, which is the common formula used to evaluate the charge. In our case, this expression is applicable to the meron in Fig. 3c but not to the Bloch point in Fig. 3b that is by nature a 3D singularity as are hopfions and skyrmion dots^24,25^. In the 3D case, the complete expression (1) must be used and the integration is over a surface of a 3D volume enclosing the singularity. We integrated over a cube and a sphere with the same surface area (cube side 84 nm and sphere diameter 116 nm) obtaining similar results. The explicit expression of the different terms of the integral is depicted in Supplementary Information.

Figure 4a shows the streamlines and arrows of emergent field obtained numerically from our reconstructed magnetization data around the Bloch point shown in Fig. 3b. The convergence of the streamlines and the directions of the auxiliary arrows provide evidence of a negative topological charge at the center (extended black volume). The total topological charge *Q* enclosed by a closed surface *S* has been evaluated from Eq. (1), integrating over the surface of a cube, as indicated in Fig. 4a. The calculated *Q* depends on the size of the cubic box *L* and on its position within the sample. *L* must be chosen so that, as mentioned above, the 3D singularity is fully enclosed in the integrated volume. Figure 4b depicts the topological charge profiles along the *x* direction for different integration cube sizes (see Supplementary Information for details). For large integration boxes, the enclosed *Q* saturates at *Q* = −1, corresponding to the charge of the Bloch point, but for a size of 42 nm it does not reach −1, indicating that the cube is too small. The effective lateral size of the Bloch point deduced from our data can be estimated by assuming that the profiles of Fig. 4b result from the convolution of a delta function (the Bloch point) with an experimental resolution function and that *Q* = 0 and *Q* = −1 correspond to the opposite limits of the Bloch point either completely inside or completely outside of the integration box. The profiles for *L* = 84 nm and 94.5 nm are very similar and enclose the full charge. The variation of *Q* from −0.1 to −0.9 extends over 22 nm as indicated by the vertical dashed lines which gives a reasonable estimate for the accuracy of the location of the magnetic singularity.

While the Bloch point displays a charge concentrated in a small 3D region, the charge of the meron shown in Fig. 3c is more spatially spread. According to its nature, the meron has been treated as a 2D singularity in the *x*, *y* plane. A map of the charge density *q*_*z*_ in that *x*, *y* plane is represented in Fig. 4c, showing dark features at the bifurcation core and noisy white/black contrast at the centers of the Bloch domain walls. The *x*, *y* integration of *q*_*z*_ over the rectangle marked with dashed lines in Fig. 4c and located at the central slice of the reconstructed volume results in *Q* = −0.44 ± 0.06. Moving the integration plane from the volume center to the centers of each top/bottom permalloy layers results in *Q* = −0.39 and *Q* = −0.33, respectively, indicating that the topological charge of the meron texture is also somewhat extended over the sample profile. The obtained value −0.44 is not very different from the expected −0.5 value for an ideal meron^33^. Moving the integration square area away from the bifurcation, on top of the straight Bloch walls, gives an oscillating *Q* = ±0.06, indicating that noise-induced fluctuations effectively average to zero away from the singularities.

In conclusion, we have shown that soft X-ray vector magnetic tomography provides a novel, relatively simple and well-suited method for determining the 3D magnetic configuration of thin films up to 200–300 nm in thickness. The method has been used to map the closure domains in Py films separated by a weak PMA ferromagnetic spacer and has allowed quantitative determination of the topological charge of 3D magnetic singularities occurring at the cores of the bifurcations. A Bloch point and meron-like singularities have been clearly identified. Further development of the technique exploiting the element sensitivity of the magnetic dichroism will allow resolving the complete configuration of the magnetization in stacks having different magnetic elements at unprecedented detail, which will provide a useful tool in a variety of heterostructures for different applications.

## Methods

### Sample preparation

The trilayer of Permalloy (Ni_80_Fe_20_) and NdCo_5_ was fabricated by DC magnetron sputtering^26^ from high purity Nd and Co targets (deposited by co-sputtering) and a Ni_80_Fe_20_ target. The used substrate was a commercial Si_3_N_4_ TEM membrane (Ted Pella, 21501-10) with 50 nm thickness and 750 μm × 750 μm size. Individual layer thicknesses of 80 nm were chosen to probe in-depth magnetization considering that the axial resolution of the tomography is ~85 nm (details in Supplementary Information) and the maximum film thickness compatible with reasonable transmission is 300–400 nm. For Py films at the Fe L_3_ resonance, Ni absorbs about six times less X rays than Fe resulting in a reasonable transmission of thick films compared to films of a pure element. The magnetic state was prepared by in-plane partial magnetization reversal from 3000 to −62 Oe, which is the magnitude of the coercive field, in order to generate relevant magnetic topological textures such as Vortex–Antivortex pairs, and meron-like configurations^20,30,31^. After fabrication and magnetic preparation, commercial Au nanoparticles (NPs) with diameters around 100 nm were deposited on the trilayer by aqueous coating. These NPs were used as fiducials for the tomogram alignment process. The solution concentration was adjusted to obtain, after drying, a particle surface density of about 8–10 particles per 100 μm^2^. The sample was subsequently loaded into the full-field X-ray transmission microscope of the Mistral beamline at the Alba synchrotron^29^ which has a high precision rotary stage.

### Microscope

The soft X-ray transmission microscope of the MISTRAL beamline at ALBA synchrotron is installed on a bending magnet beamline equipped with a variable-line-spacing grating monochromator and focusing mirror optics. The exit slits opening was 15 µm which lead to a resolving power of about 2000 (refs. ^29,39^). Circular polarized radiation was obtained by positioning an ancillary vertical slit allowing only to transmit the radiation at 0.2 mrad above or below the orbit plane of the electrons in the storage ring. After the exit slit of the monochromator, a single bounce glass capillary focused the monochromatized and polarized photons into the sample that was mounted^39^ on a set of motorized stages allowing rotation around a vertical axis. The transmitted radiation through the sample impinged a Fresnel zone plate of 25 nm outermost zone width, acting as objective lens of the microscope that operated at magnification ~1500. The transmitted image was collected by a 1024 × 1024 CCD chip with 10.5 nm effective pixel size. The whole instrument was under high vacuum.

### Data acquisition and reconstruction

Whereas in standard tomography only one tilt series normally suffices to obtain the reconstruction of the linear absorption coefficient of the sample due to the scalar nature of the electron density, in magnetic tomography it is necessary to acquire two tilt series with different sample orientations as explained in the main text. For each projection angle of both tilt series, images with opposite photon polarizations were acquired. Angular steps of 1 or 2° were used at angles above or below ±24°, respectively. The images had exposure times between 8 and 20 s to avoid saturation of the detector and exploit at its maximum the dynamic range of the detector. To improve the data quality, several images (from 15 to 20) were collected and averaged at each angle. Also, to eliminate systematic positioning errors of the goniometer, images with both polarizations were acquired sequentially at each angle.

Crucial for the accuracy of the reconstruction is the proper processing of the raw images. These were corrected and converted into transmittance images by normalizing with flat field images recorded under the same microscope conditions without any sample located between the condenser and the zone plate. The logarithms of the transmittance images were evaluated and opposite helicity images at the same angular projection were aligned using the Au fiducial NPs. These are essential for tomography of thin films since the sample shape is effectively an infinite plane and the borders cannot be used for alignment as done previously^8,13^. The magnetic contribution was isolated by performing the subtraction of positive and negative helicity images. After this, the IMOD software for projection alignment^40^ was used to construct the final data which were fed into our 3D magnetic reconstruction code^21^. Details can be found in the Supplementary Information. The fundamental principle of the reconstruction method is based on a volume model enclosing the sample of 400 × 400 × 80 voxels with voxel volume of 10.5^3^ nm^3^ and on the values of the rotation angles for all the measured projections. This allowed to calculate the X-ray path for each specific pixel in the detector passing through different voxels of the model at each rotation angle. Then, we discretized the line integral which models the experimentally recorded intensity. In this way, for each different angular orientation of the sample, the intensity in a specific pixel of the detector is expressed with a linear equation where the unknowns are the magnetization vectors. By solving the system of linear equations from all the pixels of the detector and for all the different rotation angles, we recovered the magnetization configuration within the volume model. The main difficulty with this approach is the fact that the total number of equations to be solved is well above 19 million for a detector ROI of 256 × 256 pixels and 100 different projections. Hence, Algebraic Reconstruction Techniques (ART) are necessary to solve the problem^25^. We have chosen ART since it leads to fast convergence without the necessity of any a priori knowledge of the system. The algorithm was applied to the individual tilt series and to the joint dataset, merging both. The first approach allows to reconstruct only *m*_||_ and *m* for each tilt. The second allows to merge the information contained in both tilt series together, thus reconstructing *m*_*x*_, *m*_*y*_, and *m*_*z*_ magnetization components. Both approaches were followed to observe the effects of the incomplete information due to the limited acquisition angular range (missing wedge) and the parallax influence on the reconstructed configuration.

Finally, the reconstructed images were analyzed and visualized using ImageJ^41^, Muview and Paraview programs.

## Supplementary information

Supplementary Information

## Data Availability

The data that support the findings of this study are available in Enlighten repository at the University of Glasgow (http://researchdata.gla.ac.uk/).

## Code availability

The code employed for the vector tomographic reconstruction is available from the corresponding authors upon reasonable request.
