# Supplementary material for: Revealing 3D magnetization of thin films with soft X-ray tomography: magnetic singularities and topological charges
Source: Nat Commun. 2020 Dec 14;11:6382. doi: 10.1038/s41467-020-20119-x (PMC7736288; doi:10.1038/s41467-020-20119-x)
Supplement: Supplementary file 1 — Supplementary Information [file 41467_2020_20119_MOESM1_ESM.pdf]

Supplementary Information for

Revealing 3D Magnetization of Thin Films with Soft X-ray Tomography: Magnetic Singularities and Topological Charges

A. Hierro-Rodriguez, C. Quirós, A. Sorrentino, L. M. Alvarez-Prado, J. I. Martín, J. M. Alameda, S. McVitie, E. Pereiro, M. Vélez and S. Ferrer.

Data pre-processing and tomographic projections alignment

The raw data collected at the microscope consists on sets of images acquired with clockwise (CW) and counterclockwise (CCW) photon polarizations for all the sampled different angular projections. To achieve good statistics several images were acquired at each angle. At high angles, the reduced transmission requires the acquisition of more images than at low angles. Flat field (FF) images (no sample between condenser and detector) were as well acquired for both photon polarizations to evaluate the transmittance.

The pre-processing protocol is presented in the flow chart of Supplementary Figure 1(a). The data are firstly arranged in different blocks (P blocks) comprehending the images (N_i images per block) collected for each angular projection and specific polarization (both polarizations are treated separately), as well as the FF normalization image. These are depicted as insets within each block of images in Supplementary Figure 1(a).

All images are first normalized by the FF individually, registered, and then averaged, to obtain a single transmittance image for each angular projection. As a result, a stack of images is obtained for each polarization configuration formed by all the angular projections. Secondly, CW and CCW transmittance images for each angular projection are registered by using the Au nanoparticles (NPs) as landmarks. The alignment step is very important because, as the polarization change is achieved by moving an ancillary slit to select photons emitted above and below the central plane of the electron's orbit, the illumination of the capillary condenser, which focus the light on to the sample, is slightly different for both polarizations (see Fig 1(b) in the main text). The Au fiducials are instrumental for this correction. In addition, the intensities of both polarized beams are not identical due to imperfections in the optical elements of the beamline. To correct for the XMCD contrast asymmetry, images at normal incidence for both polarizations were compared and a correction factor in the range of 1.1 to 1.2 was introduced to obtain the same magnetic contrast of the upwards and downwards magnetic domains in both polarizations. Finally, the logarithms of all corrected images were evaluated. As explained in ref. (1) the sum of the logarithms of images of both polarizations at a given angle is sensitive to the electronic charge of the sample and is not sensitive to the magnetization. On the contrary, the difference only depends on the magnetization. In our data this is not exactly true and the sum of the logarithms displays a minor contrast (about 6-7 times smaller than the XMCD contrast) arising from the striped magnetic domains. It originates from a contribution of linear dichroism since the polarization

selecting slits are relatively close (200 μrad) to the electron orbit. However, linear dichroism cancels out in the log(CW)-log(CCW) difference images due to its quadratic dependence with the magnetization.

The final step before performing the vector tomographic reconstruction consists on the alignment of the complete set of angular projections to a common rotation axis [Supplementary Fig. 1(b)]. This is performed by using the IMOD software package (2). Here, the Au fiducial nanoparticles are again crucial for achieving an accurate alignment having a direct impact on the quality of the final reconstruction.

The sum stack that exhibited intense contrast of the Au nanoparticles due to their relatively large electron density, was used for the computation of this alignment of all angular projections. We used a semi-automatic approach for selecting and tracking the Au NPs through the entire tilt series: IMOD automatic fiducial model creation and tracking tool was used and afterwards, the detected positions were optimized by the user manually and further double-checked during the fine alignment process to minimize the residuals of the global fit. This allowed locating the position of the fiducials in 3D from the IMOD model and as these are linked rigidly to the magnetic layer, the geometric transformations necessary to align the tomographic stack were calculated. Moreover, this process also allows to accurately identify the tilt angles of each projection, correcting any small inaccuracies due to the experimental set up. The calculated geometrical transformations are then directly applied to the difference stack which consists of the XMCD signal. After this, the difference aligned stack is the input of our tomographic reconstruction package (1).

Supplementary Figure 2 shows typical preprocessed transmittance images for CW and CCW polarization at several tilt angles and the corresponding log(CW/CCW) used as input data for the tomographic reconstruction.

<u>Diffraction effects in the transmitted intensity.</u>

The X-ray microscope is based in the absorption of the X-rays by the sample. In addition, scattering effects also take place and it is interesting to analyze if they contribute to the absorption images. A bending magnet photon source operated with slits as indicated in Fig 1b of the main text, has unavoidably a contamination of linearly polarized X-rays. This is much less pronounced in a beam from elliptical undulators that generate almost pure circular polarization (~70 % *vs* ~90% respectively in round numbers). As discussed by Pfau *et al.* (3), under resonant scattering conditions, linearly polarized beams generate purely magnetic diffraction patterns from domains with alternate perpendicular magnetization. Similar effects occur with circularly polarized X rays as discussed by Durr *et al.* (4). In our case, as the stripes in Fig 1b-I exhibit periodicity in one direction only, they resemble a diffraction grating generating magnetic diffracted bands with reciprocal space periodicity given by $2\pi/\Lambda = 16\ \mu m^{-1}$ which corresponds to a diffraction angle of 4.4 mrad at the photon energy of the experiments.

In our experimental set-up these effects have not to be considered since the optical architecture is completely different from those of the previous literature (3,4). In our microscope, the Fresnel zone plate that is located after the sample, acts as a lens of magnification about 1200 with a numerical aperture (NA) of approximately 0.06 which corresponds to acceptance angles from the optical axis up to 30 mrad. All rays emerging from a point in the sample within the NA of the lens will be imaged in the same point at the detector plane, i.e. un-diffracted beams (zero order) and diffracted beams at 4.4 mrad will merge in a single point in the detector plane and consequently the diffraction effects will not be observed.

Simulated reconstruction of striped domain pattern

Micromagnetic simulations of an 80nm $Ni_{80}Fe_{20}$/80nm NdCo/80 nm $Ni_{80}Fe_{20}$ have been used to calculate a tomographic reconstruction model of a striped domain pattern at the onset of magnetization reversal following the procedure described in ref. (1). Briefly, we have simulated the micromagnetic configuration of a NiFe/NdCo/NiFe multilayer with MuMax^3 (5) as described in ref. (6) (see Supplementary Fig. 3(a-b)). Next, we have computed an angular tilt series of MTXM images from the simulated micromagnetic configuration, in the same geometry as in Tilt series 1. Finally, we have used the simulated stack of projections as input data for our tomographic reconstruction method (1). The results are shown in Supplementary Fig. 3: we observe that the in-plane magnetization $m_{||1}$ presents an oscillatory contrast of short period wrinkles corresponding to the oscillation of the magnetization from out-of-plane at the stripe center to in-plane at the walls separating consecutive stripes (Supplementary Fig. 3(c-d)). This oscillation is also present in the original simulated micromagnetic configuration. The out-of-plane magnetization $m_{\perp 1}$ presents a pattern of dark/clear stripes with bifurcations (Supplementary Fig. 3(e-f)) corresponding to up/down stripe domains. Clear differences appear in $m_{||1}$ between the top and bottom reconstructed slices which are also apparent in the micromagnetic simulations (note the different top/bottom locations of blue regions in Supplementary Fig. 3(a-b) corresponding to in-plane reversed domains). These differences are caused by topological restrictions that govern the nucleation and propagation of magnetic singularities at top/bottom layers during in-plane magnetization reversal (6,7).

Closure domain structure for parallel stripe pattern. Parallax effect.

Supplementary Figure 4 displays the cross sections of the tomographic reconstruction indicated by the red arrows in the top-view images, i.e. from a plane transverse to the parallel stripes. Here, a major difference is observed between the two tilt series: the cross sections for $m_{||1}$ and $m_{\perp 1}$ show magnetic contrast extended through the whole thickness of the reconstruction volume (850nm) [Supplementary Fig. 4(c)] while $m_{||2}$ and $m_{\perp 2}$ allocate the magnetization in a confined region in z of approximately 300 nm [Supplementary Fig. 4(d)]. The reason for this difference is a parallax effect due to the different orientations of the stripes with the θ rotation axis. As visible in Fig. 1(c) (at

φ=102°), the stripes appear thinner and closer at oblique angles compared with their aspect at φ=0° (see Fig. 1(e)). In the latter case the stripes are actually much longer than the acceptance of the detector which originates the spread along z in Supplementary Fig. 4(c). These are effects of “parallax” or “apparent movement” in the recorded magnetic contrast which have paramount importance for the tomographic reconstruction. The apparent movement is small in tilt series 2 since the rotation axis is nearly parallel to the direction of the stripes. However, for tilt series 1, the rotation axis is close to be perpendicular and, in the experimental microscopy images, the stripe domains are effectively “infinite lines” since they extend over the full field of view of the microscope in the whole measured angular range. This “infinite” character hinders the spatial confinement of the reconstructed data, as shown in Supplementary Fig. 4. These differences in parallax for the two orthogonal tilt series, together with the limited angular range available due to geometrical constrains (the so-called missing wedge effect in tomographic reconstructions (8,9)) plus the shadowing effects due to the size of the membrane window and sample support are the main limitations for the accuracy of the reconstruction.

To obtain an approximate reconstruction of the magnetization vector **m** (m_x, m_y, m_z) of the periodic stripe pattern [Fig. 2(e)], we used the following procedure: ($m_x \approx m_{||1}$, $m_z = m_{\perp 1}$) and ($m_y \approx m_{||2}, m_z = m_{\perp 2}$) were obtained from the reconstruction of tilt series 1 and 2, respectively. The effect of parallax on the reconstructed data from each tilt series was estimated from the comparison between m_z components from each independent reconstruction. m_z reconstructed from tilt series 1 extends over a large z-thickness [Supplementary Fig. 4(c)], but with a reduced amplitude in comparison with m_z from tilt series 2. This is natural since, as the experimental contrast is reconstructed extending the magnetization in a thicker area, its value at each voxel is reduced. In order to obtain a qualitative image of the reconstructed magnetization components, we multiplied m_x obtained from tilt series 1 by a factor 2.5, estimated from the ratio $m_{\perp 2}/m_{\perp 1}$ evaluated near the center of the sample. The vector map of this renormalized **m** vector (Supplementary Fig. 5a) shows a very good qualitative agreement with the magnetization configuration in Supplementary Fig. 5b for a parallel stripe pattern in a $Ni_{80}Fe_{20}/NdCo_5/Ni_{80}Fe_{20}$ multilayer obtained by micromagnetic simulations with MuMax^3 (5-7).

The opposite dependences of the amplitudes of $m_\perp$ and z-confinement due to local parallax are also evident in the reconstructed data for each single tilt series near bifurcation cores. Here the stripes bend and the $m_\perp$ amplitudes increase or decrease for reconstructed data from tilt series 1 and 2 respectively. This indicates that the simultaneous reconstruction of tilt series 1 and 2 is optimum for similar parallax conditions of the domain pattern at both series of data. These conditions are met near bifurcation cores in our experiment.

Estimation of axial resolution

We took advantage of the structure of the closure domains to estimate the axial resolution (δz) achieved in the collected tomography, which depends on the measured angular range and the depth of field of the lens. The calculation of the resolution has been performed by exploiting the fact that as the sample has a thin film configuration, there are abrupt transitions above and below the top and bottom Permalloy layers. These discontinuities correspond to the interfaces Py/vacuum and Si_3N_4/Py and can be considered as step functions indicating the transitions between non-magnetic and magnetic regions. Moreover, as we know that the magnetic closure domains exhibit maximum in-plane magnetizations at the interfaces for an ideal system, we fitted Gaussian functions to the experimentally reconstructed values of $m_{||2}$ along the z direction as depicted in Supplementary Fig. 6. The sigma values of the fitted Gaussians provide a direct estimate of the resolution in the z direction resulting in $\delta z \approx 85$nm. This relatively large value compared with the x, y resolution (8), is the cause that the tomographic reconstruction of the magnetization in the central layer is not zero.

Topological Charge Calculations.

The three-dimensional topological charge was calculated following the definition as indicated in reference 10. It could be considered that a suitable choice for the closed integration surface S could be the surface of a sphere containing the singularity due to the spherical symmetry of the "emergent" field B_i^e around the Bloch point. However, the same result can be obtained with any closed surface containing the singularity (11) since the divergence of B_i^e is zero everywhere except at the singularities (12). Thus, taking into account the natural magnetization volume model arrangement in cubic voxels, the selection of a cube is the simplest approach to perform the calculation. In this framework, it is important to note that equation (1) can be split in six terms, each one corresponding to a face of the cube.

$$Q = \frac{1}{4\pi}\left[\int dSx_1\, \boldsymbol{m}\cdot\left(\frac{\partial \boldsymbol{m}}{\partial y}\times\frac{\partial \boldsymbol{m}}{\partial z}\right) + \int dSx_2\, \boldsymbol{m}\cdot\left(\frac{\partial \boldsymbol{m}}{\partial y}\times\frac{\partial \boldsymbol{m}}{\partial z}\right) + \int dSy_1\, \boldsymbol{m}\cdot\left(\frac{\partial \boldsymbol{m}}{\partial z}\times\frac{\partial \boldsymbol{m}}{\partial x}\right) + \int dSy_2\, \boldsymbol{m}\cdot\left(\frac{\partial \boldsymbol{m}}{\partial z}\times\frac{\partial \boldsymbol{m}}{\partial x}\right) + \int dSz_1\, \boldsymbol{m}\cdot\left(\frac{\partial \boldsymbol{m}}{\partial x}\times\frac{\partial \boldsymbol{m}}{\partial y}\right) + \int dSz_2\, \boldsymbol{m}\cdot\left(\frac{\partial \boldsymbol{m}}{\partial x}\times\frac{\partial \boldsymbol{m}}{\partial y}\right)\right] \quad (S1)$$

dSx_1, dSx_2, dSy_1, dSy_2, dSz_1 and dSz_2 correspond to the infinitesimal surface elements of the cube's faces with normal vectors along x, y and z directions respectively. Subscripts are associated with the sign of the face area considering 1 negative and 2 positive and **m** represents the unitary magnetization vector field. The three-dimensional topological charge within the closed integration surface is computed by calculating the spatial derivatives of the magnetization (central derivatives scheme) and performing the numerical integration. Moreover, we constructed a three-dimensional scalar map of Q across the sample by evaluating the charge within a cube of fixed dimensions centred at (x_0, y_0, z_0) (centre of the volume) that was scanned through the whole reconstructed

volume model as sketched in Supplementary Fig. 7 allowing to localize topological charges. Supplementary Figure 7a displays a top view of the calculated Q at the central sample slice with a cubic box of 84 nm lateral size, showing a clear dark region at the bifurcation core. Panels b and c display the x and z charge profiles. This method allows us to locate 3D magnetic singularities from our experimental data without any a priori assumption. A profile taken across the blue line in Supplementary Fig. 7 displays a transition from $Q = 0$ to $Q = -1$ and back to $Q = 0$ as the center of the integration box moves across the bifurcation core, depending on whether the integration box encloses the Bloch point or not. The minimum box size needed to obtain a saturated value of $Q = -1$, i.e. to fully enclose the Bloch point singularity in 3D is 84 nm. Then, the width of the transition region $-0.1 > Q > -0.9$ provides an estimate of the localization of the topological charge of the Bloch point: 22 nm for a lateral profile parallel to the x axis (Supplementary Fig. 7(b)) and 44 nm along z (Supplementary Fig. 7(c)). We have also computed the topological charge enclosed in a sphere centered at the Bloch point with the same surface area as the 84 nm side cube (i.e. 115 nm diameter) resulting in $Q = -0.95$. This confirms the expected independence of the computed topological charges on the particular shape of the integration surface.

The calculation of 2D topological charges is made by reducing eq. S1 to the integration on the z plane

$$Q = \frac{1}{4\pi}\int dSz_1\, \mathbf{m}\cdot\left(\frac{\partial \mathbf{m}}{\partial x}\times\frac{\partial \mathbf{m}}{\partial y}\right) = \int \mathbf{m}\cdot\left(\frac{\partial \mathbf{m}}{\partial x}\times\frac{\partial \mathbf{m}}{\partial y}\right) dxdy \qquad \text{(S2)}$$

which is the standard method to characterize magnetic skyrmions and merons (13,14). The integration area should cover the whole spin texture. In particular, for merons localized at stripe domain ends, it must cover the end cap in which the magnetization performs a 180º in plane rotation as shown in the theoretical analysis by Ezawa (14). Supplementary Figure 8 shows the rectangular region used for the calculation of the meron charge $Q = -0.44$ in this work on top of the reconstructed magnetization configuration at the central slice. The right border is placed to meet the 180º in-plane rotation condition. Up/right/left sides are drawn within a region of uniform out-of-plane magnetization (i.e. with zero topological charge density, as can be seen in Fig. 4(c)) so that their exact location is irrelevant. A calculation with a semicircle covering the meron area results in $Q = -0.44$, which is equivalent to the rectangular area integration.

References

1. A. Hierro-Rodriguez et al., 3D reconstruction of magnetization from dichroic soft X-ray transmission tomography. *J. Synchrotron Rad.* **25**, 1144-1152 (2018).
2. D. N. Mastronarde, S. R. Held, Automated tilt series alignment and tomographic reconstruction in IMOD. *J. Struct. Biol.* **197**, 102-113 (2017).
3. B. Pfau, *et al.*, Ultrafast optical demagnetization manipulates nanoscale spin structure in domain walls. *Nat. Commun.* **3**, 1100 (2012).

4. H. A. Durr, *et al.*, Chiral Magnetic Domain Structures in Ultrathin FePd Films. *Science* **284**, 2166 (1999).

5. A. Vansteenkiste *et al.*, The design and verification of MuMax3. *AIP Advances* **4**, 107133 (2014).

6. A. Hierro-Rodriguez *et al.*, Deterministic propagation of vortex-antivortex pairs in magnetic trilayers. *Appl. Phys. Lett.* **110**, 262402 (2017).

7. C. Quiros *et al.*, Cycloidal Domains in the Magnetization Reversal Process of $Ni_{80}Fe_{20}/Nd_{16}Co_{84}/Gd_{12}Co_{88}$ Trilayers. *Phys. Rev. Appl.* **10**, 014008 (2018).

8. J. Oton *et al.*, Characterization of transfer function, resolution and depth of field of a soft X-ray microscope applied to tomography enhancement by Wiener deconvolution. *Biomed. Opt. Express* **7**, 5092 (2016).

9. A. C. Kak, M. Slaney, *Principles of Computerized Tomographic Imaging*, (IEEE Press, New York, 1998).

10. G. E. Volovik, Linear momentum in ferromagnets, *J. Phys. C: Solid State Phys.* **20** L83 (1987)

11. P.R. Kotiuga, The algebraic topology of Bloch points. *IEEE Trans. Magn.* **25**, 3476 (1989)

12. X.S. Wang *et al.*, Current-Driven Dynamics of Magnetic Hopfions, *Phys. Rev. Lett.* **123**, 147203 (2019).

13. N. Nagaosa N and Y. Tokura Topological properties and dynamics of magnetic skyrmions. *Nat Nanotechnol.* **8,** 899-911 (2013).

14. M. Ezawa, Compact merons and skyrmions in thin chiral magnetic films. *Phys. Rev. B* **83**, 100408(R) (2011).

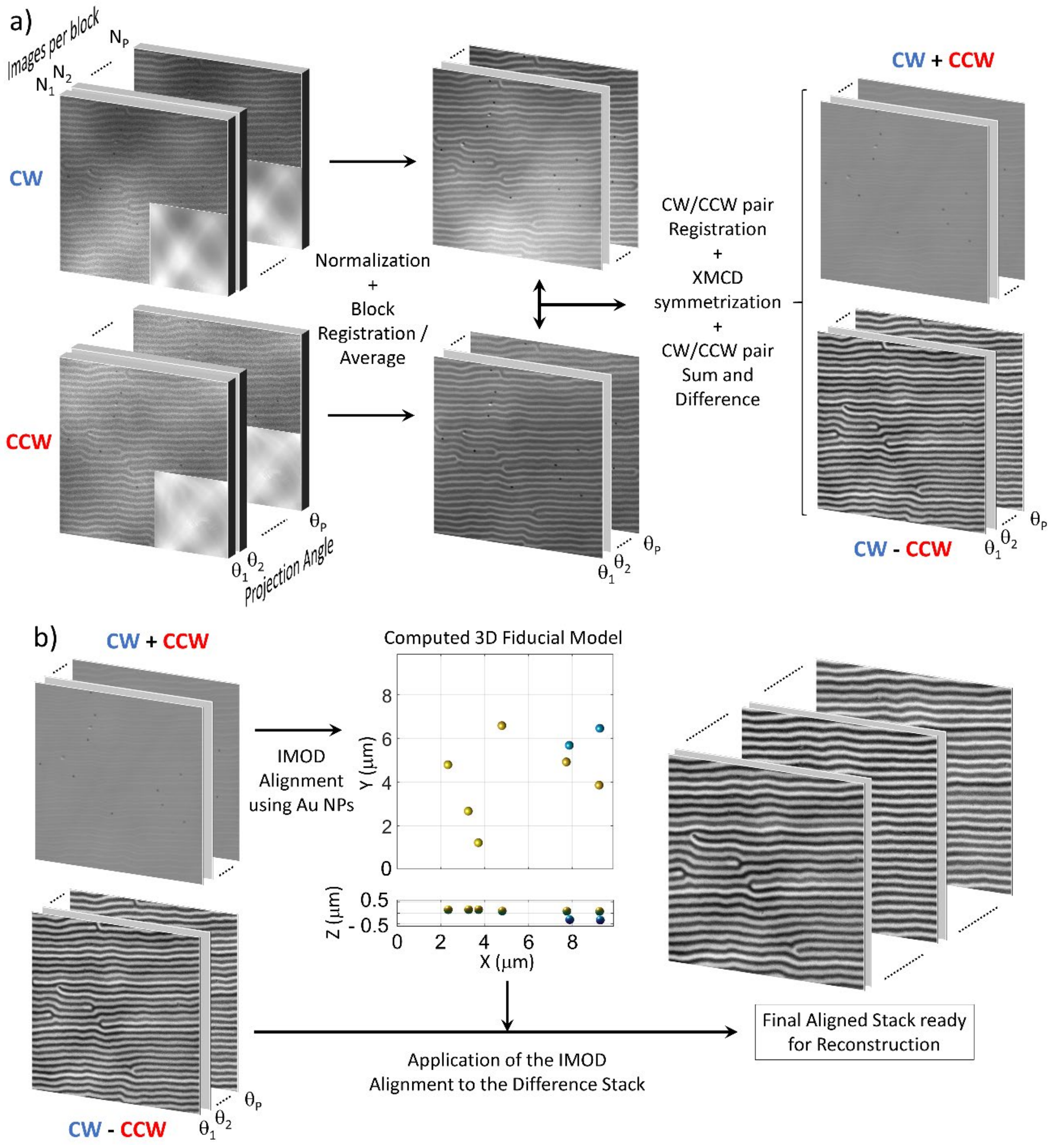

Supplementary Figure 1: Pre-processing and Tilt Series alignment flow chart. (a) Pre-processing of the raw data: i) acquisition of P blocks of N_i images at different projection angles θ_i and circular polarizations; ii) normalization, registration and averaging to form a single image for each θ_i; iii) alignment, XMCD symmetrization and logarithm calculation of images for structural/magnetic signal separation (CW+CCW and CW-CCW). (b) Flow diagram showing the combination of pre-processed stacks for the computation of the final alignment of the difference Tilt Series: i) CW+CCW images are used to compute the correct tilt angle and tomographic alignment by locating the position of the Au NPs fiducials in 3D from the IMOD model (top and side views are displayed, yellow/blue dots mark the position of Au NPs at top/bottom sides of the Si-N membrane) ii) IMOD alignment is applied to the CW-CCW stack to create the final aligned dataset containing the magnetic signal and corrected tilt angles for its introduction in the vector reconstruction algorithm.

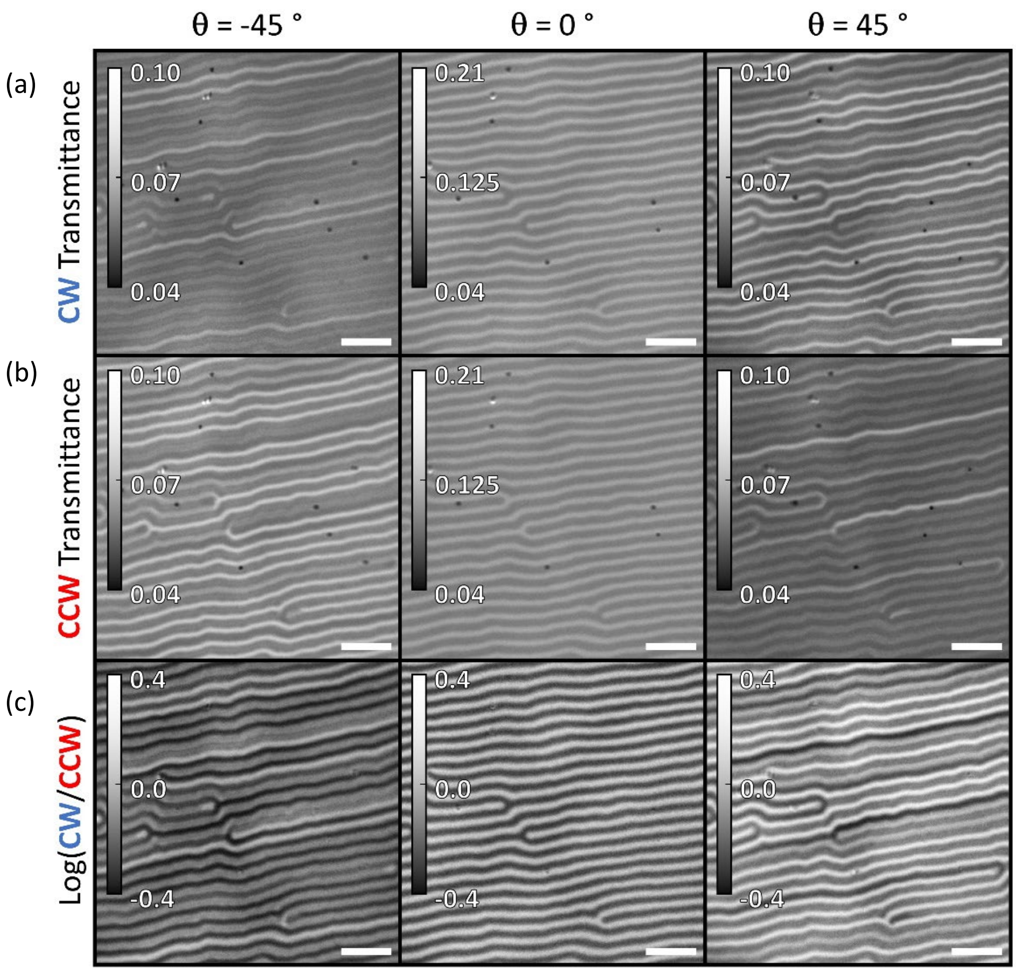

Supplementary Figure 2: Processed Transmittance and XCMD images. (a-b) CW and CCW pre-processed transmittance images at different tilt angles (45º, 0º, -45º) for Tilt series 1; (c) Final log(CW)-log(CCW) images containing the XMCD magnetic information.

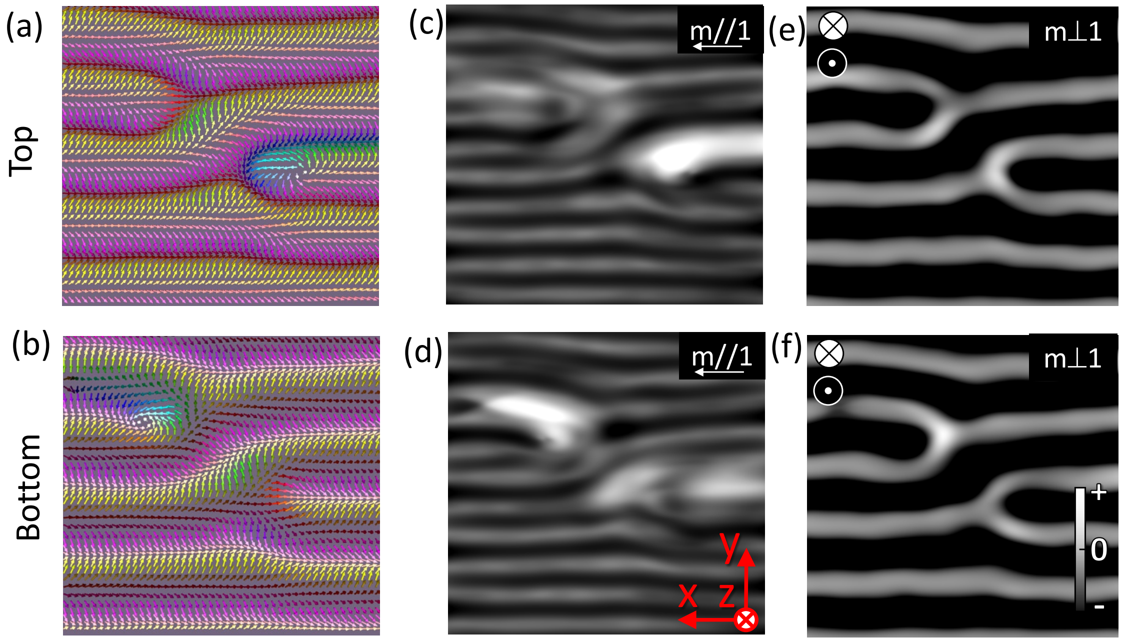

Supplementary Figure 3: Tomographic reconstruction of the magnetic configuration of an 80 nm NiFe/ 80nm NdCo/80 nm NiFe trilayer obtained from micromagnetic simulations. (a-b) Simulated micromagnetic configuration at Top and Bottom NiFe layers; (c-f) Reconstructed magnetization slices at top/bottom layers showing in-plane ($m_{||}$) and out-of-plane ($m_{\perp}$) components from simulated MTXM images in the same geometry as Tilt series 1. Note top/bottom contrast asymmetries due to the different micromagnetic configuration and the short period wrinkles in (c-d) related with domain walls separating neighboring out-of-plane stripes.

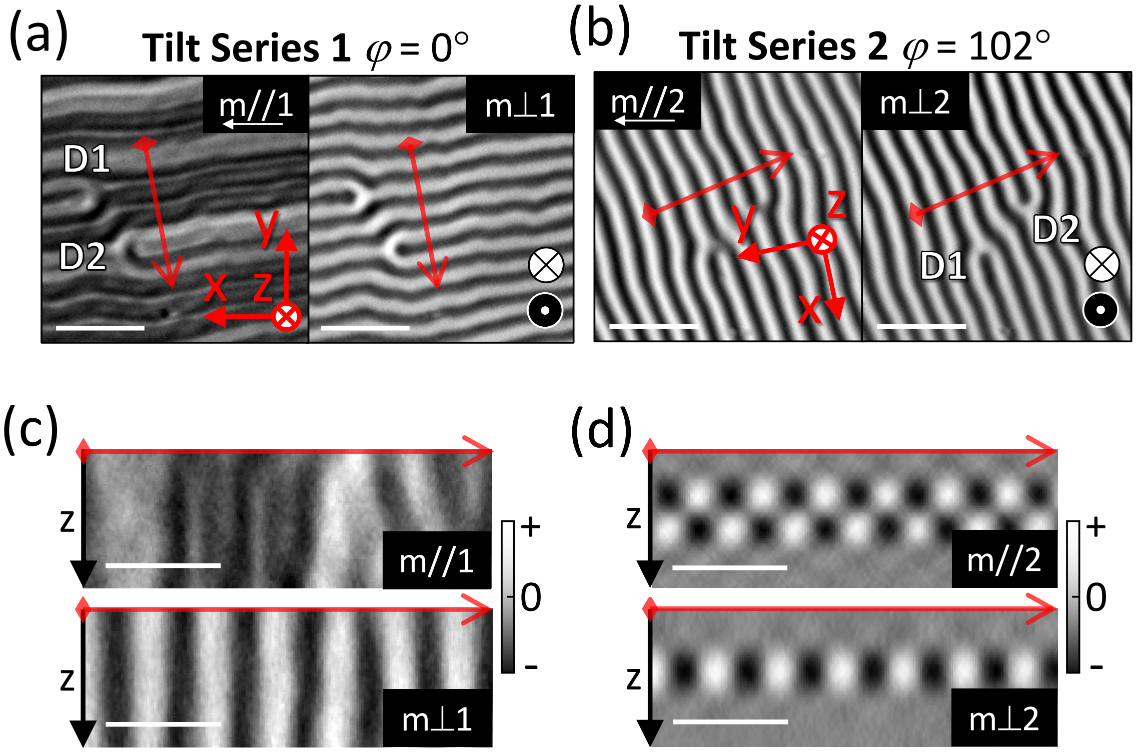

Supplementary Figure 4: In-depth cross sections of parallel striped domain pattern. (a-b) Reconstructed magnetization slices at the center of top Py layers showing in-plane ($m_{||}$) and out-of-plane ($m_{\perp}$) components for Tilt series 1 and 2. Red arrows indicate the extracted cross sections. Grey scale bars indicate the sign of the magnetization. (c-d) Cross sections of in-plane ($m_{||}$) and out-of-plane ($m_{\perp}$) components of the reconstructed magnetization for Tilt series 1 (d) and 2 (e). Scale bars (a-b) 1.4µm and (c-d) 700nm.

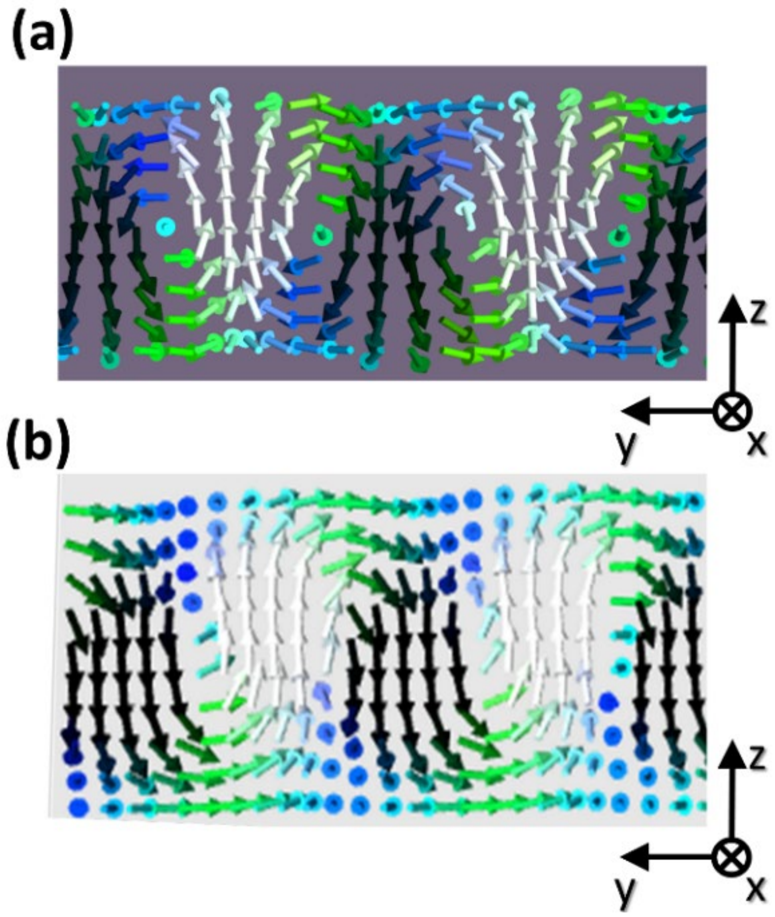

Supplementary Figure 5: Closure domain structure for parallel magnetic striped domain pattern. (a) Vector map of the reconstruction of the magnetization from Tilt series 2 (sensitive to $\boldsymbol{m_y}, \boldsymbol{m_z}$). $\boldsymbol{m_x}$ component is extracted from the reconstructed Tilt series 1 data, using a normalization factor to take into account the different parallax effects, as discussed in the text. (b) Vector map of magnetization in $Ni_{80}Fe_{20}/NdCo_5/Ni_{80}Fe_{20}$ trilayer obtained from micromagnetic simulations.

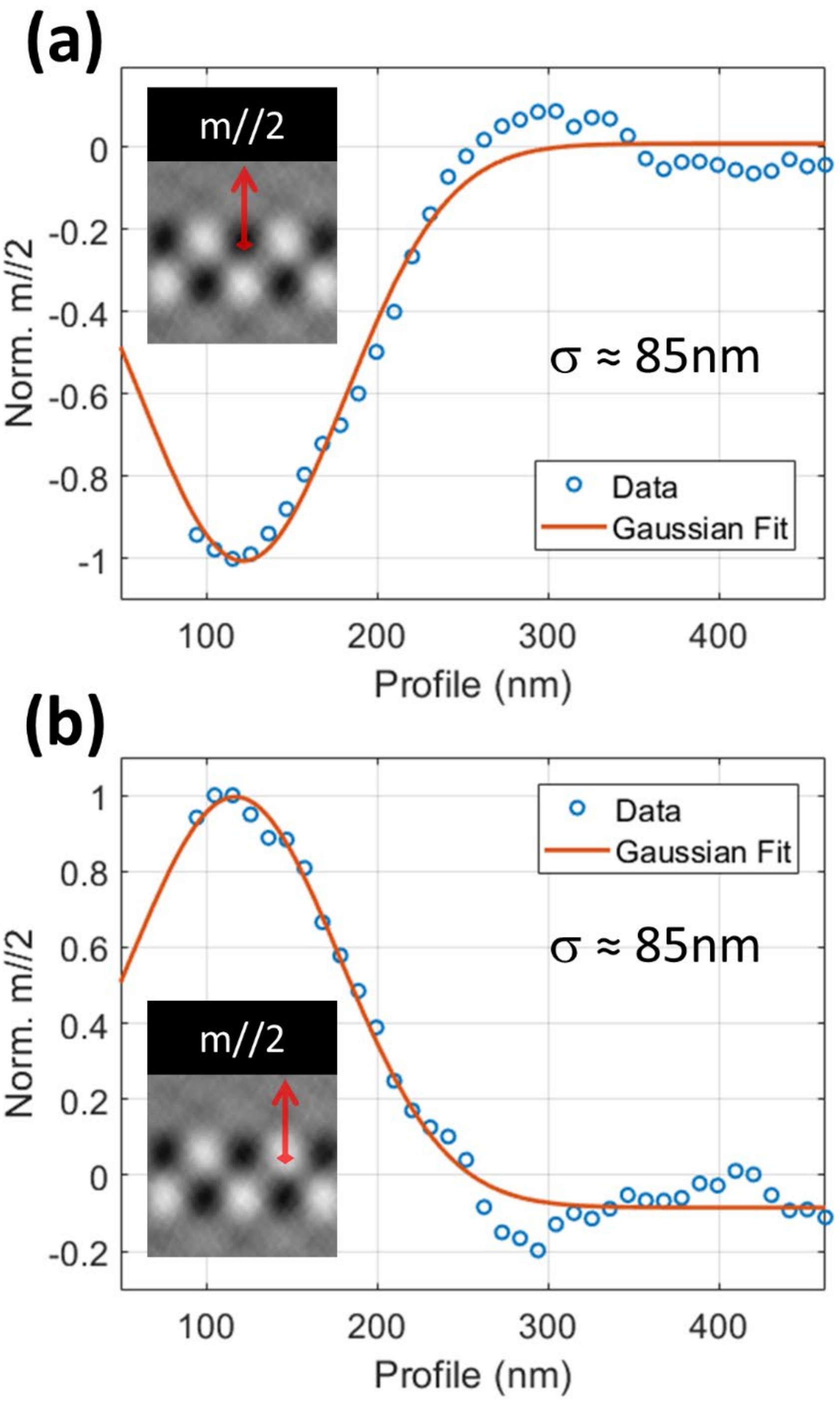

Supplementary Figure 6: Axial resolution. (a-b) Intensity profiles of $\boldsymbol{m}_{||2}$ along the $\boldsymbol{z}$ direction for negative (a) and positive (b) closure domains. The arrow in the inset figure indicates the extracted intensity profiles. A gaussian function fit has been performed and the sigma of the Gaussians are indicated and provide estimations of the axial resolution δz.

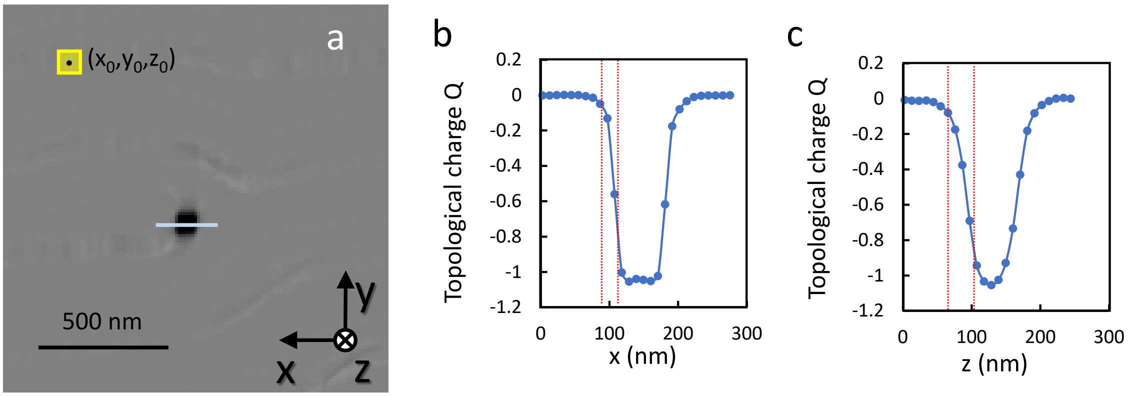

Supplementary Figure 7: Spatial distribution of total topological charge Q. (a) Top view of the topological charge enclosed by a cube of lateral size L = 84 nm (yellow box) centered at each point of the sample volume $(\boldsymbol{x_0}, \boldsymbol{y_0}, \boldsymbol{z_0})$ (black dot). Profiles taken across blue line show the sample region with largest enclosed topological charge along x (b) and z (c). The width of the transition between $\boldsymbol{Q} = \boldsymbol{0}$ and $\boldsymbol{Q} = -\boldsymbol{1}$ (red dashed lines) gives an estimate of the localization of the Bloch point since it provides the value of the lateral displacement of the integration box required to switch between an "empty box" and a "Bloch point fully inside the box" configurations.

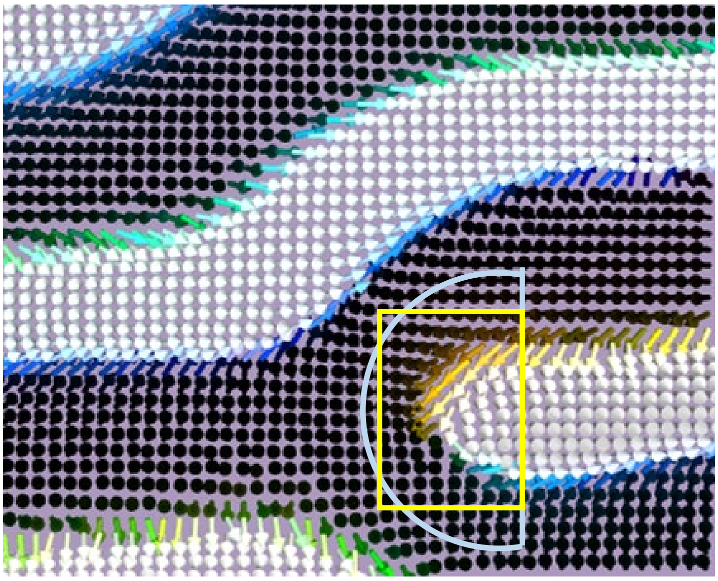

Supplementary Figure 8. Top view of reconstructed magnetization configuration at the central slice of the sample near D2. Yellow rectangle and blue semicircle indicate the integration region for eq. S2 used to calculate the topological charge of the meron localized at the end of the stripe domain.
